# Impact of Ischemic Preconditioning on Outcome in Clinical Liver Surgery: A Systematic Review

**DOI:** 10.1155/2015/370451

**Published:** 2015-02-10

**Authors:** Michael J. J. Chu, Ryash Vather, Anthony J. R. Hickey, Anthony R. J. Phillips, Adam S. J. R. Bartlett

**Affiliations:** ^1^Department of Surgery, University of Auckland, Private Bag 92019, Auckland 1142, New Zealand; ^2^Maurice Wilkins Centre for Biodiscovery, University of Auckland, Private Bag 92019, Auckland 1142, New Zealand; ^3^School of Biological Sciences, University of Auckland, Private Bag 92019, Auckland 1142, New Zealand; ^4^New Zealand Liver Transplant Unit, Auckland City Hospital, Private Bag 92024, Auckland 1023, New Zealand

## Abstract

*Background*. Ischemia-reperfusion injury is a major cause of post-liver-surgery complications. Ischemic preconditioning (IPC) has been demonstrated to protect against ischemia-reperfusion injury. Clinical studies have examined IPC in liver surgery but with conflicting results. This systematic review aimed to evaluate the effects of IPC on outcome in clinical liver surgery. *Methods*. An electronic search of OVID Medline and Embase databases was performed to identify studies that reported outcomes in patients undergoing liver surgery subjected to IPC. Basic descriptive statistics were used to summarise data from individual clinical studies. *Results*. 1093 articles were identified, of which 24 met the inclusion criteria. Seven topics were selected and analysed by subgroup. There were 10 studies in cadaveric liver transplantation, 2 in living-related liver transplantation, and 12 in liver resection. IPC decreases hepatocellular damage in liver surgery as determined by transaminases but does not translate to any significant clinical benefit in orthotopic liver transplant or liver resection. *Conclusions*. Available clinical evidence does not support routine use of IPC in liver surgery as it does not offer any apparent benefit in perioperative outcome. Further clinical studies will need to be carried out to determine the subset of patients that will benefit from IPC.

## 1. Introduction

Ischemia-reperfusion injury (IRI) is a pathophysiological phenomenon where cellular damage is caused by reperfusion and reoxygenation following ischemia [1]. IRI is a major cause of morbidity and mortality following liver resection and transplantation [2, 3]. The liver can be subjected to various types of IRI [3]. This most notably includes warm IRI where inflow occlusion of the portal triad is applied during hepatic resection to decrease blood loss and in the setting of rewarming IRI where a donor liver is reperfused after prolonged cold storage during orthotopic liver transplantation (OLT). Severe IRI can lead to liver failure or death [1, 2]. In liver surgery, hepatic steatosis has been associated with worse outcome and it is hypothesized that this is because steatotic livers are less tolerant to IRI [4, 5]. In OLT, moderate-severe (>30%) hepatic steatosis of the donor organ is associated with increased graft failure [6, 7]. Similarly, patients with >30% hepatic steatosis are reported to have increased morbidity following liver resection [8, 9]. Surgeons can expect to encounter steatotic livers with increased frequency as this reflects the corresponding global “metabolic syndrome” epidemic [10, 11].

A number of strategies have been developed to attenuate the deleterious physiological effect of IRI in liver surgery, including ischemic preconditioning (IPC). IPC was first described in a renal [12] and, subsequently, emulated in a cardiac model [13]. Both studies demonstrated that a brief initial period of ischemia followed by reperfusion (“preconditioning”) prior to a period of prolonged ischemia led to improved functional outcomes. In the case of the liver, the effect of IPC was first described in 1993 in an experimental rat model [14], with improved rat survival and liver function in the rodents subjected to IPC prior to 90 minutes of ischemia. The clinical benefit of IPC in liver resection was first translated to humans in 2000 and importantly these salutary effects were seen to occur in steatotic liver resections as well [15]. IPC was described in clinical OLT in 2005 but did not decrease graft injury [16]. However, the authors only applied 5 minutes of ischemia during IPC in contrast to other studies which demonstrated that 10 minutes of ischemia was required for the beneficial effect of IPC [17].

Since the initial publication of the efficacy of IPC in hepatic steatosis subjected to IRI [15], the protective effect of IPC on steatotic livers has been the topic of research for several laboratories. Due to the proven experimental [18, 19] and clinical [15, 20] advantages, IPC has remained an important strategy in attenuating the impact of IRI on steatotic livers and has remained a readily applicable technique in clinical practice.

Previous systematic reviews [21–23] have focused on randomized control trials (RCT) and so overlook useful observations from many of the other studies published in the literature that are case-control, retrospective, or nonrandomized prospective studies. Currently, there is no cohesive literature reference resource overview of the outcomes of IPC following IRI in clinical liver resection and OLT in patients with hepatic steatosis.

The aim of this study was to systematically review the literature and provide a concise description of the impact of IPC on liver resection and liver transplantation in humans, with special emphasis on outcomes in steatotic livers.

## 2. Methods

A systematic electronic search was conducted through the OVID Medline and Embase databases from inception to February 2014 according to the Preferred Reporting Items for Systematic Reviews and Meta-analyses (PRISMA) guidelines. A combination of keyword searches (.mp) and MeSH terms (/) was used as follows: (*ischaemic preconditioning.mp *OR* ischemic preconditioning.mp *OR* Ischemic preconditioning/*) AND (*liver.mp* OR* hepatic.mp *OR* Liver/*OR* steatosis.mp* OR* exp Fatty liver/*). Identified articles were limited to the English language. For this study, IPC was defined as the application of a brief period of ischemia and reperfusion prior to a prolonged ischemic insult [20].

The inclusion criteria were for articles investigating the use of IPC in humans undergoing* liver transplantation* or* liver resection*. This included study designs that were randomized or nonrandomized trials, prospective observational studies, retrospective reviews, and case-control studies. Articles were excluded if they were not original research (systematic review, narrative review, commentary, or editorial) or did not report clinically relevant outcomes (e.g., graft or recipient survival, histological findings or liver functions tests). Studies of living-related liver transplantation (LRLT) recipients were included in the group of OLT but results from the donor hepatectomy were excluded as these patients were not subjected to prolonged ischemia during liver transection.

Two reviewers (Michael J. J. Chu, Ryash Vather) independently executed searches, using titles and abstracts to manually screen through identified articles. Study eligibility was determined using a standardized* pro forma*, with subsequent data extraction to an Excel spreadsheet. Discrepancies were adjudicated by the senior author (Adam S. J. R. Bartlett). Duplicates and publications with overlapping study populations were excluded (the full text with the largest number of subjects was included). A manual search of the reference lists from included articles was conducted to identify any other potentially relevant studies. Information extracted from each publication were study population, duration of IPC, type of hepatic surgery, duration and type of ischemia (warm/cold), severity and type of steatosis, liver function tests (LFT), histology, duration of hospital stay, duration of intensive care unit (ICU) stay, postoperative morbidity, graft survival, and patient survival. Qualitative assessment of included articles was not performed as the aim of this review was to present outcomes from all published clinical studies. Systematic reviews investigating randomized trials exclusively have been published elsewhere [21, 22] and this was not the purpose of this current study. For studies with incomplete study details or outcome measures, the corresponding author was contacted via e-mail for additional data. If data was presented graphically, the author was contacted for numerical values and if these were not available, data were measured using digital image analysis software (ImageJ; http://imagej.nih.gov/ij/) [24, 25].

Basic descriptive statistics were used to summarise data pooled from individual clinical studies. Figures and tables were used where appropriate to facilitate ease of interpretation. No comparative statistical analyses or tests of significance were planned or undertaken. Results are shown as mean ± standard error of mean (SEM).

## 3. Results

**Figure 1:**
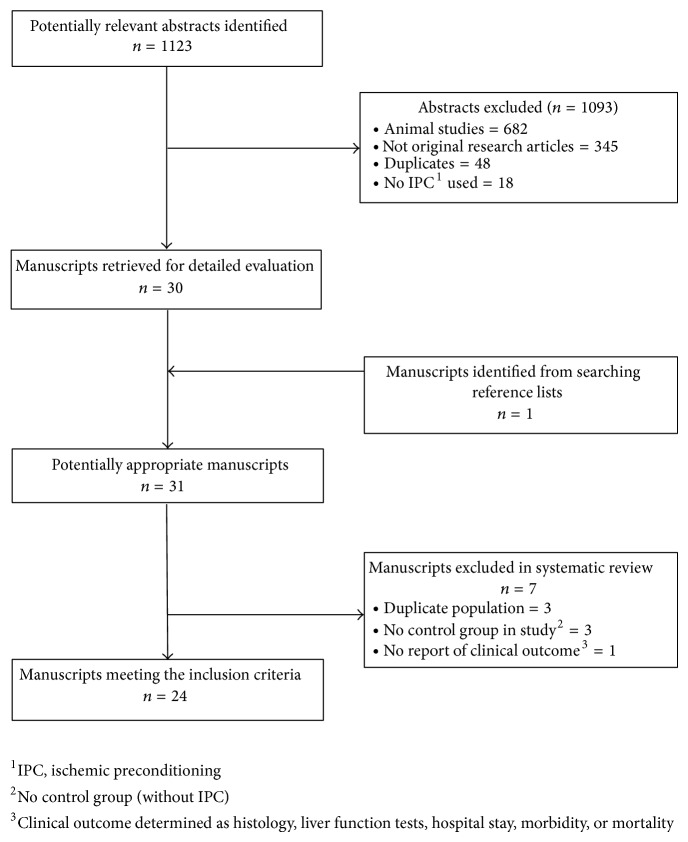
Quorum diagram.

A total of 507 and 616 articles were identified in Medline and Embase, respectively. After the exclusion of duplicates, 1075 abstracts were screened and 30 manuscripts were obtained for further evaluation. One additional manuscript was identified from searching the journal article bibliographies. Twenty-four manuscripts met all inclusion criteria and formed the basis of this study (Figure 1). The impact of IPC in OLT (Tables 1 and 2) and liver resection (Tables 3 and 4) was examined in 12 studies each. There was no delay/interval between the IPC stimulus and the IRI episode in all 24 studies.

### 3.1. Overview of Studies

In the 12 studies of IPC in OLT, there were 6 RCT, 4 prospective studies, 1 case-control study, and 1 retrospective analysis (Tables 1 and 2). Two studies examined the impact of IPC in LRLT (Table 1).

In the 12 studies of IPC in liver resection, there were 6 RCT, 4 prospective studies, 1 case-control study, and 1 retrospective analysis (Tables 3 and 4).

Six of the 24 studies investigated the impact of IPC on outcome in steatotic livers with three studies focused on recipients of donor steatotic livers (Table 5), and the remaining three studies were of patients with hepatic steatosis that underwent liver resection (Table 6).

Two articles were excluded as they were written by the same group of authors in the same hospital during a similar time period [26, 27]. Although it was not specified, it was assumed that they described the results from the same cohort of patients who were more fully reported in the publication that was included in our review [28].

### 3.2. Topic 1: Nonrandomized Studies of Ischemic Preconditioning in Cadaveric Orthotopic Liver Transplantation (Table 1)

#### 3.2.1. Study Descriptions

Four nonrandomized studies reported the effect of IPC in 101 liver transplant recipients while 103 received a nonpreconditioned liver graft (Table 1). The type of donor was reported as donation after brain death in all cases. Two studies used an IPC protocol of 10 minutes of ischemia and 10 minutes of reperfusion (10 + 10) while another study used 10 minutes of ischemia with 15 minutes of reperfusion (10 + 15) and the remaining study used 10 minutes of ischemia with reperfusion of up to 30 minutes. The mean total pretransplant ischemic time reported in the studies ranged from 360 to 660 minutes. Outcome measures reported included LFT (*n* = 4 studies), histology (*n* = 2), morbidity (*n* = 4), hospital stay (*n* = 2), ICU stay (*n* = 4), graft survival (*n* = 1), and patient survival (*n* = 2).

#### 3.2.2. Biochemistry

Posttransplantation LFT status was reported in all 4 studies. Peak aspartate aminotransferase (AST) was observed to be lower in recipients of preconditioned livers (mean 489 versus 838 IU/L) in all 4 studies but was only statistically significant in 3/4 studies [29–31] while no statistical difference was observed in the remaining study [32]. Peak alanine aminotransferase (ALT) was also observed to be lower in recipients of preconditioned livers (mean 412 versus 717 IU/L) in the 3 studies that reported levels of ALT and was significant in 2/3 studies [30, 31]. No statistical difference was observed in the remaining study [32]. Peak total bilirubin levels were described in 3 studies and no difference was observed between the groups in all 3 studies [30–32]. Coagulation profile (prothrombin time, PT, or international normalized ratio, INR) was reported in 3 studies and demonstrated similar findings between the groups [29, 31, 32].

#### 3.2.3. Histology

Postreperfusion histology was reported in 2 studies. Assessment of histological injury based on necrotic indices (lobular, periportal, and perivenous areas) was used in 1 study [30] and the remaining study assessed the severity of IRI based on presence of >10% hepatocyte necrosis [31]. Preconditioned liver grafts demonstrated decreased histological damage scores and necrosis in both studies and this was statistically significant in 1 study [31].

#### 3.2.4. Operative and Postoperative Outcomes

Intraoperative blood loss was reported in 1 study and the preconditioned group demonstrated a trend towards increased mean intraoperative blood loss (4661 versus 3686 mL) [29]. Perioperative blood product transfusion was reported in 3 studies and no difference in average amount of transfusion was observed between the two groups [30–32]. The duration of ICU stay was reported in all 4 studies with a mean of 11.9 and 10.3 days in the IPC and control group, respectively. One study observed a significantly shorter ICU admission in the IPC group [29], while 2 studies observed no difference [30, 32] and the remaining study observed a trend towards an increased duration of ICU stay [31]. The duration of hospital stay was reported in 2 studies with a mean of 38 and 31 days in the IPC and control group, respectively. One study observed no difference [30], whereas 1 study demonstrated a trend towards increased duration of hospital stay in the IPC group [31]. Postoperative complications were reported in 2 studies and no difference was observed between the IPC (28%) and control (22%) group [30, 31].

Postoperative graft dysfunction was reported as initial poor function (IPF) or primary nonfunction (PNF) in 2 studies. Both studies observed no difference in rates of PNF between IPC (0%) and non-IPC (1.5%) groups [31, 32]. One study observed increased rates of IPF in the IPC group (33% versus 13%) [31], whereas the remaining study showed a trend towards increased IPF in the IPC group (11% versus 0) [32]. Three of the 4 studies described the incidence of acute rejection and it was lower in the IPC group (19% versus 36%) but was not statistically significant in all 3 studies [29–31]. Only one study described the incidence of chronic rejection and there was a trend towards a decreased incidence of it in the IPC group (8% versus 38%) [30]. Graft survival was reported in one study with similar 1-year outcome between IPC (89%) and non-IPC liver grafts (90%) [32]. Patient survival was reported in 2/4 studies and demonstrated no difference in 1-year patient survival between the IPC (94%) and non-IPC (96%) groups [31, 32].

#### 3.2.5. Conclusion

In nonrandomized studies of IPC in OLT, the use of IPC was associated with lower increase of liver transaminases and decreased histological injury but had similar levels of postoperative bilirubin and coagulation profiles as those of nonpreconditioned liver grafts. Despite decreased biochemical and histological injury markers in the IPC group, preconditioned liver grafts had similar perioperative outcome as nonpreconditioned liver grafts. IPC was associated with a trend towards decreased rates of acute and chronic rejection but, paradoxically, there was a trend towards increased rates of IPF compared to nonpreconditioned liver grafts.

### 3.3. Topic 2: Studies of Ischemic Preconditioning in Living-Related Liver Transplantation (Table 1)

Two prospective nonrandomized studies reported the effect of IPC in 32 living-related liver transplant recipients and 32-matched recipients of nonpreconditioned liver graft. Both studies used 10 + 10 minutes as the IPC protocol. The mean total ischemic time ranged from 120 to 155 minutes. Outcome measures reported included graft survival (*n* = 2), patient survival (*n* = 2), morbidity (*n* = 2), hospital stay (*n* = 2), ICU stay (*n* = 1), histology (*n* = 1), and LFT (*n* = 2). Histological assessment of IRI was based on presence of >10% hepatocyte necrosis. Based on these two studies, there was no difference in all outcome measures between recipients of IPC and nonpreconditioned liver grafts [33, 34].

### 3.4. Topic 3: Randomized Control Trials of Ischemic Preconditioning in Orthotopic Liver Transplantation (Table 2)

#### 3.4.1. Study Descriptions

Six RCT reported the effect of IPC in 206 liver transplant recipients while 194 received a nonpreconditioned liver graft. The type of donor was reported as donation after brain death in all cases. Three studies used 10 minutes of ischemia with a reperfusion phase up to 30 minutes as IPC, while one study used 10 + 10 minute and one used 10 + 15 minute IPC. The remaining study used 5 minutes of ischemia with continuous reperfusion up to the time of portal triad clamping (Table 2). The mean total ischemic time ranged from 376 to 580 minutes. Outcomes reported included graft survival (*n* = 5), patient survival (*n* = 4), morbidity (*n* = 6), hospital stay (*n* = 4), ICU stay (*n* = 1), histology (*n* = 4), and LFT (*n* = 6).

#### 3.4.2. Biochemistry and Histology

Posttransplantation LFT were reported in all 6 studies. Peak ALT in the IPC group was 524 IU/L, compared to 691 IU/L in the control group. Two of the 5 studies reported decreased ALT [35, 36], whereas two studies reported increased ALT in the IPC group [16, 37] and the remaining study observed no difference [38]. Peak AST was also observed to be lower in recipients of preconditioned liver grafts (639 versus 960 IU/L) but this finding was observed in 3/6 studies [35, 36, 39] and 2 studies observed increased AST in the IPC group [16, 37] while the remaining study observed no difference [38]. Peak total bilirubin levels were described in all 6 studies and there was no difference between the groups. There was also no difference in INR and PT levels between the groups in all 6 studies. Postreperfusion histology was reported in 4/6 studies with variable histological assessment criteria for IRI between all 4 studies. Three of four studies had similar findings between the groups [16, 36, 37] while one study observed decreased hepatocyte swelling in the IPC group [38].

#### 3.4.3. Operative and Perioperative Outcomes

Intraoperative blood loss was reported in 1 study and there was no difference between the groups [36]. Perioperative blood product transfusion was observed to be similar between the IPC and control group in 5 studies. The duration of ICU stay was reported in 1 study and the IPC group had a similar mean ICU stay of 6.8 days compared to 6.7 days in the control group [36]. The duration of hospital stay was reported in 4/6 studies with a mean of 13.2 and 15.1 days in the IPC and non-IPC group, respectively. All 4 studies showed no difference between the 2 groups. Postoperative complication was reported in 1 study and there was a trend towards decreased rate of complications in the IPC group (12 versus 22%) [36].

IPF was reported in 2 studies and both observed no difference in rates of IPF between the IPC (10%) and non-IPC (13%) group. Five studies reported on rates of PNF and observed no difference between the IPC (2%) and non-IPC (3%) group in 4/5 studies. The remaining study observed a trend towards decreased rate of PNF in the IPC group [36]. Incidence of acute rejection was reported in 4 studies with a mean rate of 20.8% and 20.9% in the IPC and non-IPC group, respectively. Three of the 4 studies observed no difference while one study observed a trend towards decreased rate of moderate-severe acute rejection in the IPC group [37]. Graft survival was reported in 5 studies with 6-month, 1-year, and 2-year rates of 93%, 91%, and 78% in the IPC group and 89%, 82%, and 74% in the non-IPC group, respectively. Patient survival was reported in 4 studies with 6-month, 1-year, and 2-year rates of 91%, 100%, and 85% in the IPC group and 82%, 92%, and 76% in the non-IPC group, respectively. There was no difference in graft or patient survival between the 2 groups.

#### 3.4.4. Conclusions

In RCT of IPC in OLT, the use of IPC had variable results with respect to LFT measurements and there was no effect on bilirubin, coagulation profile, or histological findings. Consistent with biochemical and histological findings, there was no significant difference in operative or perioperative outcome between preconditioned and nonpreconditioned liver grafts.

### 3.5. Topic 4: Nonrandomized Studies of Ischemic Preconditioning in Liver Resection (Table 3)

#### 3.5.1. Study Descriptions

Six nonrandomized studies reported the impact of IPC in 125 patients that underwent liver resection compared to 122 patients that did not receive IPC. Five of the six studies excluded liver cirrhosis in their study population and the remaining study exclusively investigated the impact of IPC in cirrhotic patients [40]. Four studies used 10 + 10 minute IPC, while one study used 10 + 15 minute and the remaining study used 5 minute of ischemia and 5 minute of reperfusion (5 + 5). Five of the 6 studies utilized Pringle maneuver [41] and the remaining study used total vascular exclusion (Table 3). The mean warm ischemic time ranged from 18 to 45 minutes. Outcomes reported included patient mortality (*n* = 4), morbidity (*n* = 6), hospital stay (*n* = 5), ICU stay (*n* = 4), histology (*n* = 1), and LFT (*n* = 6).

#### 3.5.2. Biochemistry and Histology

Postoperative LFT were reported in all 6 studies. Peak ALT was reported in 5/6 studies with mean levels of 244 IU/L in the IPC group compared to 413 IU/L in the non-IPC group. Four of the 5 studies observed significantly lower peak ALT levels in the IPC group and the remaining study observed no difference between the groups [42]. Peak AST was reported in all 6 studies with mean levels of 223 and 502 IU/L in the IPC and non-IPC group, respectively. Five of the 6 studies reported significantly lower peak AST levels in the IPC group and the remaining study observed no difference in AST levels between the groups [42]. Coagulation profile was reported in 4 studies and there were no significant difference between the groups. Total bilirubin was reported in 4 studies and was observed to be lower in only one study [40] whereas the remaining studies did not observe any difference between the groups. Postreperfusion histological necrosis was demonstrated to be significantly lower in patients with prior IPC in one study [42].

#### 3.5.3. Operative and Perioperative Outcomes

Intraoperative blood loss was reported in 4 studies and there were no differences between the groups. Blood product transfusion was reported in 3 studies and was observed to be significantly lower in the IPC group in one study [15] whereas the remaining two studies did not observe any difference [43, 44]. The duration of ICU stay was described in 4 studies and the IPC group had a similar mean ICU stay compared to the control group in all 4 studies (1.6 versus 2 days). Duration of hospital stay was reported in 5/6 studies with a mean of 11.8 and 13.7 days in the IPC and non-IPC group, respectively. Four of the 5 studies found no difference between the group and one study reported decreased hospital stay in the IPC group [40]. Postoperative complications were reported in 4 studies and three studies found no significant difference between the groups, whereas one study observed significantly lower rates of major complications in the IPC group [15]. There was no perioperative mortality in all 6 studies.

#### 3.5.4. Conclusions

In nonrandomized studies of IPC in liver resection, IPC was associated with attenuation of liver injury as measured by liver transaminases and histology but there was no effect on postoperative bilirubin and coagulation profile. Despite biochemical and histological improvement, IPC had no effect on perioperative outcomes.

### 3.6. Topic 5: Randomized Control Trials of Ischemic Preconditioning in Liver Resection (Table 4)

#### 3.6.1. Study Descriptions

Six RCT reported the impact of IPC in 207 patients that underwent liver resection, with 209 patients that did not receive IPC. All six studies excluded liver cirrhosis in their study population. Five studies used 10 + 10 minute IPC and the remaining study used 10 + 15 minute IPC. Two studies used total vascular exclusion or intermittent portal triad clamping, whereas the remaining 2 studies used continuous Pringle maneuver. The mean warm ischemic time ranged from 34 to 49 minutes. Outcomes reported included patient mortality (*n* = 5), morbidity (*n* = 6), hospital stay (*n* = 5), ICU stay (*n* = 5), and LFT (*n* = 6).

#### 3.6.2. Biochemistry

Postoperative peak ALT was reported in 5/6 studies. Two studies found no difference in peak ALT levels between the groups [45, 46] whereas one study observed a trend towards increased ALT [47] and another observed a trend towards decreased ALT in the IPC group [48]. The remaining study reported significantly lower peak ALT in the IPC group [20]. Peak AST was reported in 4/6 studies. Two studies observed significantly lower levels of AST in the IPC group [20, 49] and one observed similar levels between the two groups [45], whereas the remaining study reported a trend towards increased AST in the IPC group [47]. Postoperative coagulation profile and total bilirubin were reported in 5 and 6 studies, respectively. All studies reported no significant difference in postoperative coagulation profile or total bilirubin between the two groups.

#### 3.6.3. Operative and Perioperative Outcomes

Intraoperative blood loss was reported in all 6 studies with mean IOBL of 691 and 729 mL in the IPC and non-IPC group, respectively. Five of the 6 studies reported no difference between the groups and the remaining study observed significantly lower blood loss in the IPC group [48]. Perioperative blood product transfusion was reported in all 6 studies. Consistent with the findings of intraoperative blood loss, 5/6 studies observed no significant difference in blood product transfusion and one study observed a significantly lower amount of perioperative transfusion in the IPC group [48]. The duration of ICU admission was reported in 5/6 studies with a mean of 2 and 2.5 days in the IPC and non-IPC group, respectively. Four of the 5 studies observed no difference in ICU admission between the groups and one study observed a trend towards a decreased duration of ICU admission in the IPC group [47]. Hospital stay was also reported in 5/6 studies with a mean of 12.2 and 12.4 days in the IPC and non-IPC group, respectively. Four of the 5 studies reported no difference between the 2 groups whereas one study observed a trend towards decreased hospital stay in the IPC group [47]. Postoperative complications were reported in all 6 studies and mean total complication rates were 40% in the IPC group and 48.9% in the non-IPC group. Five of the 6 studies reported no difference in postoperative complications between the groups and the remaining study observed a significantly lower rate of complications in the IPC group (20% versus 45%) [48]. Perioperative mortality was reported in all 6 studies with a mean perioperative mortality of 1.4% and 1.9% in the IPC and non-IPC group, respectively. All 6 studies reported no difference in rates of perioperative mortality and 3/6 studies had no perioperative mortality [20, 45, 49].

#### 3.6.4. Conclusions

In RCT of IPC in liver resection, there was no significant difference in all outcome measures (biochemically or perioperatively) between the IPC and non-IPC groups. There were variable results in measurements of liver transaminases but other biochemical and perioperative outcomes were more consistent in demonstrating no difference between the two groups.

### 3.7. Topic 6: Impact of Ischemic Preconditioning on Outcome of Steatotic Livers in Orthotopic Liver Transplantation (Table 5)

#### 3.7.1. Study Descriptions

Ten of the 12 studies documented outcomes in 201 recipients of steatotic donor liver grafts and 99 of the 201 recipients received a preconditioned steatotic liver. From these 201 recipients, a total of 54 recipients from 3 studies were analyzed as a subgroup with 25 and 29 recipients in the IPC and control group, respectively [16, 30, 38]. Two of the 3 studies were a RCT and the remaining study was a retrospective study (Table 5). One study categorized the presence of any steatosis to the steatotic group [30] whereas the degree of steatosis was categorized as >15% macrovesicular in one study [38] and there was no documentation of the severity of steatosis in the remaining study [16]. The type of steatosis was reported in all 3 studies, with 2 documenting macrovesicular, and the remaining study had a predominance of mixed hepatic steatosis [30]. All studies were of recipients of donor after brain death liver grafts. The protocol of IPC was 10 + 10 minutes in one study [30] and 10 + 30 minutes in another study [38] while the remaining study performed IPC immediately after laparotomy [16]. The mean total ischemic time in these studies ranged from 437 to 518 minutes. Outcome measures reported included LFT (*n* = 3), histology (*n* = 1), morbidity (*n* = 1), ICU (*n* = 1), hospital stay (*n* = 1), and graft survival (*n* = 1).

#### 3.7.2. Biochemistry, Histology, and Perioperative Outcomes

Postoperative peak ALT and AST were described in three [16, 30, 38] and two [30, 38] studies, respectively. One study described significantly higher levels of ALT in the IPC group [16] whereas the other 2 studies observed no difference in ALT levels between the groups. Peak AST levels were observed to be significantly lower in the IPC group in one study [38], whereas the remaining study observed no difference [30]. Coagulation profile and total bilirubin were reported in two studies and both studies observed no significant difference between the groups [30, 38]. Histological necrosis was reported in one study with decreased necrosis in the IPC group [30]. Clinical outcome (hospital stay, ICU, morbidity, and perioperative transfusion) were similar between the groups [30]. There was no difference in 6-month graft survival [38] but preconditioned steatotic liver grafts were associated with decreased rates of acute and chronic rejection [30].

#### 3.7.3. Conclusions

In studies of effect of IPC on steatotic livers in OLT, there was a lack of accurate description of severity of hepatic steatosis but this may be due to small numbers of liver grafts with hepatic steatosis in the study. Preconditioned steatotic livers may be associated with lower histological injury and rates of rejection but there was no difference in biochemical or other clinical outcome measures between the two groups.

### 3.8. Topic 7: Impact of Ischemic Preconditioning on Outcome of Steatotic Livers in Liver Resection (Table 6)

#### 3.8.1. Study Descriptions

Seven of the 12 studies documented outcomes in 104 patients with hepatic steatosis that underwent liver resection. From these 104 patients, a total of 29 patients from three studies were analyzed as a subgroup with 16 and 13 patients in the IPC and non-IPC group, respectively [15, 20, 49]. Two studies were RCT and the remaining study was a prospective nonrandomized study [15]. The subgroups were <30 or >30% steatosis but the type of steatosis was not described in all three studies. The protocol of IPC was 10 + 10 minutes prior to continuous Pringle maneuver in two studies [15, 20] and 10 + 15 minutes prior to total vascular exclusion in the remaining study [49]. Mean warm ischemic time ranged from 30 to 42 minutes. Postoperative levels of ALT and AST were compared in one and three studies, respectively. IPC was associated with lower levels of ALT [15] and AST [15, 20, 49] in steatotic livers. No other outcome measures were compared.

#### 3.8.2. Conclusions

There were only small numbers of patients with hepatic steatosis in studies investigating IPC in liver resection and there was a lack of description of the type of steatosis. Preconditioning was associated with a decrease in transaminases but there were no other outcome measures described.

## 4. Discussion

IRI is a multifactorial process that plays an often unavoidable major role in liver damage during liver surgery [1, 2]. Although there are no current therapeutic options to prevent IRI [50], multiple protective strategies have been proposed and one strategy routinely used in clinical practice is IPC. IPC was first described in the 1980s and involves repeated episodes of brief ischemia [12, 13]. IPC conferred surprising protection against subsequent IRI [51] and was first reported in clinical practice by Clavien et al. [15], who then proposed that IPC may protect steatotic livers from IRI. As steatotic livers are more susceptible to IRI [5], IPC could provide an attractive and simple strategy to attenuate the impact of IRI. However, multiple clinical studies over the last decade presented conflicting results of the effects of IPC in liver resection and OLT [21, 22]. The evidence from this review suggests that while IPC appears to confer a protective effect in decreasing signs of hepatocellular damage (decreased transaminases), this does not translate to significant clinical benefit as measured by hospital stay, complication rates, or survival.

The studies referred to in this review have used various durations of IPC with the majority of studies except two [16, 40] utilizing 10 minutes of ischemia in their IPC protocol. However, the reperfusion phase of the IPC varied from 10 to 39 minutes in studies of OLT, whereas conversely studies of liver resection were more consistent (10–15 minutes). Despite the variability in IPC protocol, Glanemann et al. demonstrated that 30–45 minutes of reperfusion was more effective than 5–15 minutes in an experimental warm ischemia setting [52]. Additionally, the expected clinical variability in the duration of ischemia in the studies combined with the various IPC protocol may potentially be contributing to the discrepancy of results seen. Future studies should standardize the protocol of IPC to minimize interstudy variability and allow improved comparison between studies.

### 4.1. Improvement in LFT Did Not Translate to Clinical Outcome Benefit

While IPC associates with decreased serum transaminase after transplantation and liver resection, this finding is inconsistent. In liver transplantation, the decrease in hepatocellular injury was not associated with improved liver synthetic function or histological findings as one would expect. Similarly, the discrepancy of findings in studies of liver resection may be due to the different mechanism in the methods of vascular control used. Furthermore, the improvement in hepatocellular injury was not associated with an improvement in morbidity or mortality in studies of liver transplantation and resection. A recent review suggested that the benefit of IPC is proportional to the severity of IRI [53]. This may explain the discrepancy between improvement of liver function tests but with no associated improvement in clinical outcome. The majority of patients in clinical studies of IPC in liver resection were subjected to such a short duration of warm ischemia; it was not considered a severe or lethal injury. Similarly, a large study of liver transplantation has shown that the majority of posttransplantation recipients have mild reperfusion injury [54]. It is possible that the already low rates of morbidity and PNF in studies of liver resection and transplantation, respectively, coupled with relatively small patient numbers, which meant that the studies were insufficiently powered to detect differences with respect to clinical outcomes [22]. Future studies of IPC should include higher risk patients for liver resection (hepatic steatosis or cirrhosis) and liver transplantation (high donor risk index [55]) to allow us to further delineate which patient group may best benefit from IPC.

### 4.2. IPC in Living-Related Liver Transplantation

There have been only two studies of IPC in LRLT published in the literature thus far [33, 34]. There is a need to consider the unique setting of LRLT with short ischemic time and healthier donors who have no underlying liver disease compared to cadaveric liver transplantation. These liver grafts would be categorized as low donor risk index grafts [55] and would be associated with low adverse outcomes. The conclusion from these two studies is that IPC in this setting was not associated with any beneficial effect but, importantly, IPC was not associated with adverse outcomes in recipients or donors. Donor safety and sufficient graft function are both paramount in LRLT, and although IPC appears safe it may not have any utility in LRLT [33].

### 4.3. IPC in Steatotic Livers

The deleterious effects of IRI on steatotic livers have been documented experimentally [56, 57] and clinically [9, 58]. The impact of IPC on steatotic livers has also been documented experimentally [18, 59], but data from clinical studies remains scarce. The first study to describe the impact of IPC on outcome in patients with hepatic steatosis was in 2000 [15]. Since then, five other studies have performed subgroup analysis of the impact of IPC on patients with hepatic steatosis in OLT [16, 30, 38] and liver resection [20, 49]. While these studies have shown a potential beneficial effect of IPC in patients with hepatic steatosis, outcomes were available for only a small number of patients (54 in OLT and 29 in liver resections). In the studies of OLT, one study performed a subgroup analysis on marginal donors defined as age >65 years and/or presence of >15% macrovesicular steatosis [38], whereas one study did not describe the severity of steatosis [16]. As age is an important factor in the effect of IPC [20, 53], age may have also played a role in the study outcome [38]. In the studies of liver resection, the type of steatosis was not documented and this may have confounded the results as the beneficial effect of IPC may potentially be greater in microvesicular steatosis [19]. Future studies of hepatic steatosis will require detailed descriptions of type and severity of steatosis to allow comparison between studies. Additionally, a large number of patients will be required to provide statistical robustness in determining the efficacy of IPC in steatotic livers.

### 4.4. Missing Subgroups from Liver Resection

Currently, there is no strong evidence to support or refute the routine use of IPC in both liver resection and liver transplantation. However, there needs to be further research into the effect of IPC in certain population subgroups that were routinely excluded from clinical studies such as patients with underlying chronic liver disease (such as hepatic steatosis or cirrhosis), previous liver resection, or hepatic chemoembolisation/radiofrequency ablation. There has been only one prospective nonrandomized study of IPC in patients with cirrhosis [40] and it showed that IPC may potentially be beneficial in this patient population. There has as yet been no further publication to replicate the findings of that study. As patients with underlying liver disease are more susceptible to IRI [5], it is important to find strategies to attenuate liver injury in this group of patients to improve outcome and patient safety. Similarly, patients with previous liver resection or hepatic chemoembolisation/radiofrequency ablation also require further investigations on the efficacy of IPC in this patient population. Furthermore, outcome measures (both laboratory and clinical) should be standardized between studies to facilitate comparison. To further delineate which subgroup of patients will benefit from IPC, a multicentre prospective randomized trial is likely to be required. This is particularly important for patients with hepatic steatosis as there will be an ever-increasing incidence of hepatic steatosis encountered by liver surgeons [10].

## 5. Conclusions

This review has demonstrated that IPC decreases hepatocellular damage in liver surgery as determined by transaminases, but this does not translate to any significant clinical benefit. Importantly, findings from nonrandomized studies were consistent with findings from randomized trials in both OLT and liver resection, suggesting that the results were not biased by nonrandomization of patients. In the subgroup analysis of patients with hepatic steatosis and undergoing either liver resection or OLT, IPC appears to decrease hepatocellular damage but does not appear to have a clinical benefit. Based on this review, there is no clear indication for routine use of IPC in liver surgery, as there is no significant clinical benefit demonstrated for patients undergoing liver surgery. However, given its apparent safety, further larger studies in an RCT setting would help determine if there are any patient subgroups that would benefit from IPC.

## Figures and Tables

**Table 1:** Summary of outcome in nonrandomized clinical studies of ischemic preconditioning in orthotopic liver transplantation.

Author	Year	Study type	Donor type	IPC	No IPC	Duration of IPC (mins)	Mean ischemic time (mins)	Outcome measures	Effect of IPC
Jassem et al. [29]	2006	Case control	DBD	9	14	10 + up to 30	690	Blood tests, AR, AST, ICU, INR, IOBL, PNF	↓AR (NS), ↓AST, ↓ICU, ↑IOBL (NS)

Cescon et al. [32]	2009	Prospective^1^	DBD	20	20	10 + 15	360	Bili, ICU, GS, IPF, INR, LFT, PNF, PS, transfusion	No difference

Esposti et al. [30]	2011	Retrospective	DBD	26	24	10 + 10	440	AR, Bili, CR, Histo, HS, ICU, LFT, morbidity, transfusion	↓ALT/AST^2^

Azoulay et al. [31]	2005	Prospective^3^	DBD	46	45	10 + 10	448	AR, Bili, Histo, HS, ICU, IPF, LFT, morbidity, PNF, PS, PT, transfusion	↓ALT/AST, ↑HS/ICU (NS), ↓necrosis, ↑IPF

Andreani et al. [33]	2010	Prospective	LRLT	22	22	10 + 10	155	AR, Bili, GS, Histo, HS, ICU, LFT, morbidity, PNF, PS, PT, transfusion	No difference

Testa et al. [34]	2010	Prospective	LRLT	10	10	10 + 10	120	AR, Bili, GS, HS, INR, LFT, morbidity, PS, transfusion	No difference

ALT, alanine aminotransferase; AR, acute rejection; AST, aspartate aminotransferase; Bili, bilirubin; CR, chronic rejection; DBD, donation after brain death; GS, graft survival; Histo, histology; HS, hospital stay; ICU, intensive care unit stay; INR, international normalized ratio; IOBL, intraoperative blood loss; IPC, ischemic preconditioning; IPF, initial poor function; LFT, liver function tests; LRLT, living-related liver transplantation; NS, no statistically significant difference according to the author; PNF, primary nonfunction; PS, patient survival; PT, prothrombin time.

^1^IPF not defined.

^2^In nonsteatotic allografts only.

^3^IPF defined as minimal PT <30% normal level and/or maximum bilirubin >200 *μ*mol/L in absence of hemolysis or biliary obstruction.

**Table 2:** Summary of outcome in randomized controlled trials of ischemic preconditioning in orthotopic liver transplantation.

Author	Year	Donor type	IPC	No IPC	Duration of IPC (mins)	Mean ischemic time (mins)	Outcome measures	Effect of IPC
Koneru et al. [37]	2007	DBD^1^	50	51	10 + up to 39	410	AR, Bili, GS, Histo, HS, INR, IPF, LFT, PNF, PS, transfusion	↑ALT/AST, ↓moderate-severe AR (NS)

Jassem et al. [39]	2009	DBD	19	16	10 + 27–34	580	AR, AST, Bili, INR	↓AST

Franchello et al. [38]	2009	DBD	30	45	10 + 30	518	AR, Bili, GS, Histo, HS, INR, LFT, PNF	↓Hepatocyte swelling

Cescon et al. [35]	2006	DBD^2^	23	24	10 + 15	385	Bili, GS, IPF, LFT, PNF, PS, PTA, transfusion	↓ALT/AST

Amador et al. [36]	2007	DBD	30	30	10 + 10	376	AR, Bili, GS, Histo, HS, ICU, IOBL, LFT, morbidity, PNF, PS, PT, transfusion	↓ALT/AST, ↓PNF (NS)

Koneru et al. [16]	2005	DBD	34	28	5 + on-going reperfusion	437	GS, Histo, LFT, PS, PNF, transfusion	↑ALT/AST (NS)

ALT, alanine aminotransferase; AR, acute rejection; AST, aspartate aminotransferase; Bili, bilirubin; DBD, donation after brain death; GS, graft survival; Histo, histology; HS, hospital stay; ICU, intensive care unit stay; INR, international normalized ratio; IOBL, intraoperative blood loss; IPC, ischemic preconditioning; IPF, initial poor function; LFT, liver function tests; NS, no statistically significant difference according to the author; PNF, primary nonfunction; PS, patient survival; PT, prothrombin time; PTA, prothrombin activity.

^1^IPF defined as INR >3.0 and/or total Bili >15 mg% in absence of biliary obstruction.

^2^IPF defined as minimal PTA <30% normal level and/or maximum Bili >15 mg/dL in absence of hemolysis or biliary obstruction.

**Table 3:** Summary of outcome in nonrandomized clinical studies of ischemic preconditioning in liver resection.

Author	Year	Study type	IPC	No IPC	Duration of IPC (mins)	Mean ischemic time (mins)^1^	Outcome measures	Effect of IPC
Theodoraki et al. [43]	2011	Case control	21	21	10 + 15	44	AST, HS, ICU, IOBL, morbidity, transfusion	↓AST

Domart et al. [42]	2009	Retrospective	31	30	10 + 10	45 (TVE)	Bili, Histo, HS, ICU, IOBL, LFT, PT	↓Necrosis

Choukér et al. [28]	2005	Prospective	25	24	10 + 10	35	HS, ICU, LFT, PT	↓ALT/AST

Nuzzo et al. [44]	2004	Prospective	21	21	10 + 10	45	Bili, LFT, morbidity, PTA, transfusion	↓ALT/AST

Clavien et al. [15]	2000	Prospective	12	12	10 + 10	30	Bili, HS, ICU, IOBL, LFT, morbidity, PT, transfusion	↓ALT/AST, ↓transfusion requirement, ↓major postoperative complications

Li et al. [40]	2004	Prospective^2^	15	14	5 + 5	18	Bili, HS, IOBL, LFT, morbidity	↓ALT/AST, ↓Bili, ↓HS, ↓postoperative complications

ALT, alanine aminotransferase; AST, aspartate aminotransferase; Bili, bilirubin; Histo, histology; HS, hospital stay; ICU, intensive care unit stay; IOBL, intraoperative blood loss; IPC, ischemic preconditioning; LFT, liver function tests; PT, prothrombin time; PTA, prothrombin activity; TVE, total vascular exclusion.

^1^Continuous Pringle maneuver unless otherwise specified.

^2^Patients with liver cirrhosis only.

**Table 4:** Summary of outcome in randomized controlled trials of ischemic preconditioning in liver resection.

Author	Year	IPC	No IPC	Duration of IPC (mins)	Mean ischemic time (mins)^1^	Outcome measures	Effect of IPC
Arkadopoulos et al. [49]	2009	41	43	10 + 15	42 (TVE)	AST, Bili, HS, ICU, IOBL, PT, morbidity, transfusion	↓AST

Azoulay et al. [47]	2006	30	30	10 + 10	46 (TVE)	Bili, HS, ICU, IOBL, LFT, morbidity, PT, transfusion,	↑ALT/AST (NS), ↓HS/ICU (NS)

Winbladh et al. [45]	2012	16	16	10 + 10	42 (IPTC)	Bili, HS, INR, IOBL, LFT, morbidity, transfusion	No difference

Scatton et al. [46]	2011	40	39	10 + 10	49 (IPTC)	ALT, Bili, HS, ICU, IOBL, morbidity, PT, transfusion,	No difference

Heizmann et al. [48]	2008	30	31	10 + 10	34	ALT, Bili, ICU, IOBL, morbidity, transfusion	↓ALT (NS) ↓IOBL, ↓postoperative complications, ↓transfusion requirement

Clavien et al. [20]	2003	50	50	10 + 10	36	Bili, HS, ICU, IOBL, morbidity, LFT, PT, transfusion	↓ALT/AST

ALT, alanine aminotransferase; AST, aspartate aminotransferase; Bili, bilirubin; HS, hospital stay; ICU, intensive care unit stay; IOBL, intraoperative blood loss; IPC, ischemic preconditioning; IPTC, intermittent portal triad clamping; LFT, liver function tests; NS, no statistically significant difference according to the author; PT, prothrombin time; TVE, total vascular exclusion.

^1^Continuous Pringle maneuver unless otherwise specified.

**Table 5:** Outcome in clinical studies of ischemic preconditioning in orthotopic liver transplantation with subgroup analysis of hepatic steatosis.

Author	Year	Study type	Donor type	IPC	No IPC	% steatosis (Type)	Duration of IPC (mins)	Mean ischemic time (mins)	Outcome measures	Effect of IPC
Franchello et al. [38]	2009	RCT	DBD^1^	4	9	>15% (MaS)	10 + 30	518	Bili, GS, INR, LFT	↓AST, ↑GS (NS)

Esposti et al. [30]	2011	Retrospective	DBD	12	10	0–60% (mixed)	10 + 10	440	AR, Bili, CR, Histo, HS, ICU, LFT, morbidity, PT, transfusion	↓AR, ↓CR, ↓necrosis

Koneru et al. [16]	2005	RCT	DBD	9	10	Not stated (MaS)	5 + on-going reperfusion	437	ALT	↑ALT

ALT, alanine aminotransferase; AR, acute rejection; AST, aspartate aminotransferase; Bili, bilirubin; CR, chronic rejection; DBD, donation after brain death; GS, graft survival; Histo, histology; HS, hospital stay; ICU, intensive care unit stay; INR, international normalized ratio; IPC, ischemic preconditioning; LFT, liver function tests; MaS, macrovesicular steatosis; NS, no statistically significant difference according to the author; PT, prothrombin time; RCT, randomized control trial.

^1^Analyzed as group of marginal donor grafts (marginal donor defined as >15% MaS and/or age >65).

**Table 6:** Outcome in clinical studies of ischemic preconditioning in liver resection with subgroup analysis of hepatic steatosis.

Author	Year	Study type	IPC	No IPC	% steatosis (Type)	Duration of IPC (mins)	Mean ischemic time (mins)	Outcome measures	Effect of IPC
Arkadopoulous et al. [49]	2009	RCT	5	4	>30% (not stated)	10 + 15	42 (TVE)	AST	↓AST

Clavien et al. [20]	2003	RCT	7	6	>25% (not stated)	10 + 10	36	AST	↓Peak AST (363 versus 602 UI/L)

Clavien et al. [15]	2000	Prospective	4	3	>25% (not stated)	10 + 10	30	ALT, AST	↓ALT/AST (<260 UI/L each patient at day 1)

ALT, alanine aminotransferase; AST, aspartate aminotransferase; IPC, ischemic preconditioning; RCT, randomized control trial; TVE, total vascular exclusion.
